# Upregulation of CENPM is associated with poor clinical outcome and suppression of immune profile in clear cell renal cell carcinoma

**DOI:** 10.1186/s41065-023-00262-3

**Published:** 2023-01-13

**Authors:** Zhi-Cheng Zhang, Yi-Fu Liu, Ping Xi, Ye-Chen Nie, Ting Sun, Bin-Bin Gong

**Affiliations:** Department of Urology, The First Affiliated Hospital of Nanchang University, Nanchang, 330000 Jiangxi Province China

**Keywords:** Centromere protein M, Clear cell renal cell carcinoma, Poor prognosis, Immune suppression

## Abstract

**Background:**

The response of advanced clear cell renal cell carcinoma (ccRCC) to immunotherapy is still not durable, suggesting that the immune landscape of ccRCC still needs to be refined, especially as some molecules that have synergistic effects with immune checkpoint genes need to be explored.

**Methods:**

The expression levels of CENPM and its relationship with clinicopathological features were explored using the ccRCC dataset from TCGA and GEO databases. Quantitative polymerase chain reaction (qPCR) analysis was performed to validate the expression of CENPM in renal cancer cell lines. Kaplan-Meier analysis, COX regression analysis and Nomogram construction were used to systematically evaluate the prognostic potential of CENPM in ccRCC. Besides, single gene correlation analysis, protein–protein interaction (PPI) network, genetic ontology (GO), kyoto encyclopedia of genes and genomes (KEGG) and gene set enrichment analysis (GSEA) were used to predict the biological behaviour of CENPM and the possible signalling pathways involved. Finally, a comprehensive analysis of the crosstalk between CENPM and immune features in the tumor microenvironment was performed based on the ssGSEA algorithm, the tumor immune dysfunction and exclusion (TIDE) algorithm, the TIMER2.0 database and the TISIDB database.

**Results:**

CENPM was significantly upregulated in ccRCC tissues and renal cancer cell lines and was closely associated with poor clinicopathological features and prognosis. Pathway enrichment analysis revealed that CENPM may be involved in the regulation of the cell cycle in ccRCC and may have some crosstalk with the immune microenvironment in tumors. The ssGSEA algorithm, CIBERSOPT algorithm suggests that CENPM is associated with suppressor immune cells in ccRCC such as regulatory T cells. The ssGSEA algorithm, CIBERSOPT algorithm suggests that CENPM is associated with suppressor immune cells in ccRCC such as regulatory T cells. Furthermore, the TISIDB database provides evidence that not only CENPM is positively associated with immune checkpoint genes such as CTLA4, PDCD1, LAG3, TIGIT, but also chemokines and receptors (such as CCL5, CXCL13, CXCR3, CXCR5) may be responsible for the malignant phenotype of CENPM in ccRCC. Meanwhile, predictions based on the TIDE algorithm support that patients with high CENPM expression have a worse response to immunotherapy.

**Conclusions:**

The upregulation of CENPM in ccRCC predicts a poor clinical outcome, and this malignant phenotype may be associated with its exacerbation of the immunosuppressive state in the tumor microenvironment.

**Supplementary Information:**

The online version contains supplementary material available at 10.1186/s41065-023-00262-3.

## Introduction

Renal cell carcinoma (RCC) is a cancer that originates in the renal epithelium and includes more than 10 histological and molecular subtypes, of which clear cell RCC (ccRCC) is the most common [1]. Although patients with early stage ccRCC can be cured radically by surgical or ablative strategies, approximately one third will eventually develop metastatic disease [2]. There is no doubt that advanced ccRCC relies heavily on systemic therapy, and in particular immune checkpoint inhibitors (ICIs) -based strategies have emerged as a first-line treatment option [3]. However, despite the encouraging success of ICIs, resistance to these drugs has limited the number of patients who can achieve durable responses [4]. Indeed, this is currently the greatest challenge for immunotherapy in ccRCC. The molecular landscape associated with immune checkpoints in ccRCC therefore needs to be continually refined to help us better understand the molecular crosstalk in the tumor immune microenvironment.

Centromere protein M (CENPM), also known as proliferation-associated nuclear element 1 (PANE1), was originally detected in mouse mammary epithelial cells [5]. It not only affects the cell cycle by regulating chromosome segregation during cell division, but also encodes a new histocompatibility antigen in B lymphocytes that is involved in the immune response [6, 7]. Furthermore, the upregulation of CENPM in human cancer tissues has been found to be associated with certain malignant phenotypes. For instance, upregulation of CENPM expression can promote hepatocarcinogenesis through a variety of mechanisms, and also to some extent influence the progression of pancreatic as well as lung adenocarcinoma [8–10]. At the same time, several other genes of the centromere protein family have been found to be closely associated with tumor development, such as CENPA [11], CENPE [12], and CENPF [13]. Clearly, this also supports the link between CENPM and cancer aggressiveness to a certain extent.

Intriguingly, with the help of RNA sequencing data from the TCGA database, we found that the mRNA expression of CENPM was significantly upregulated in ccRCC tissue. However, whether it has some effect on the malignant behaviour of ccRCC or on the survival outcome of patients remains unclear. Here, therefore, we first validated the upregulation of CENPM through other public datasets and renal cancer cell lines, and then analysed the association of its expression with the prognosis of ccRCC patients. Further, with the help of gene set enrichment analysis (GSEA), we explored its potential pathway of action in ccRCC.

## Methods

### Access to RNA sequencing data and clinical information

RNA sequencing data were downloaded from the TCGA (https://portal.gdc.cancer.gov/) and GEO databases(https://www.ncbi.nlm.nih.gov/gds/), which contain paired and unpaired samples. ccRCC patients’ clinicopathological data (age, sex, clinical and pathological stage, histological grading, etc.) and prognostic information were obtained from the TCGA database.

### Quantitative polymerase chain reaction (qPCR) analysis

After extraction of total RNA from the cell lines (HK-2, 769-P, ACHN and 786-O), reverse transcription and qPCR were performed using cDNA synthesis kits (Qiagen, USA) and SYBR real-time PCR kits (Qiagen, USA) according to the kit’s instructions; quantitative analysis was based on the 2-ΔΔCt method. The primer sequences are as follows: CENPM_F: GCGGACTCGATGCTCAA; CENPM_R: GATTCACACTGGAGGGCAA; the internal reference gene is β-actin.

### UALCAN database

UALCAN (2022; http://ualcan.path.uab.edu/) is a comprehensive, user-friendly, interactive web resource that allows users to identify biomarkers or perform in silico validation of potential genes of interest [14]. Here, it is used to assess the epigenetic regulation of CENPM expression by promoter methylation.

### Nomogram construction and evaluation

Indicators with independent prognostic value screened in multivariate COX analysis were included in the construction of the Nomogram to predict disease-specific survival (DSS) in ccRCC patients at 1, 3 and 5 years; predictive efficacy was evaluated by calibration curves drawn by the “rms “R package.

### Protein–protein interaction (PPI)

The top 300 genes most strongly associated with CENPM and differentially expressed genes from the TCGA database (logFC> 1.5) were cross-tabulated and target molecules were subsequently subjected to PPI network construction to find key genes associated with CENPM in ccRCC. PPI networks were constructed in the STRING database (version:11.5; https://cn.string-db.org/) and subsequently imported into Cytoscape (version 3.9.1) for embellishment.

### Genetic ontology (GO), Kyoto encyclopedia of genes and genomes (KEGG) and GSEA

Differential genes associated with CENPM were used for GO and KEGG pathway analysis to explore the biological processes that may be involved in CENPM in ccRCC. GSEA has been shown to be a pathway enrichment analysis algorithm that controls type I and type II errors well, leading to widespread use in the processing of multi-omics data [15]. The analysis was performed using the ‘clusterprofiler’ in the R package; normalised enrichment scores (NES) > 1, false discovery rates (FDR) < 0.25 and adjusted *p*-values < 0.05 were considered statistically significant.

### Single-sample GSEA (ssGSEA) and TIMER 2.0

ssGSEA is an extension of the GSEA method and was originally designed to compensate for the inability to do GSEA on a single sample [16]. TIMER v2.0 (http://timer.comp-genomics.org/) is also a comprehensive resource that allows users to explore the full range of tumor immunological, clinical and genomic features [17]. In this study, these was used to analyse the correlation between CENPM with the abundance of immune infiltrating lymphocytes in the tumor microenvironment.

### TISIDB databases

TISIDB (http://cis.hku.hk/TISIDB/) is a web portal for exploring tumor and immune system interactions that integrates multiple heterogeneous data types [18]. We used this platform to systematically analyse the correlation between CENPM with immune checkpoint inhibitors and chemokines in ccRCC.

### Immunohistochemistry (IHC)

The specific steps are consistent with our previous studies [19]. In brief, paraffin sections were dewaxed, rehydrated, placed in sodium citrate and then heated in a microwave for antigen retrieval. Sections were then blocked with 1% BSA and incubated overnight at 4 °C with primary antibodies (anti-CENPM, anti-PD-L1). Subsequently, sections were incubated with secondary antibodies for 1 hour at 37 °C, stained with diaminobenzidine and counterstained with hematoxylin. Finally, images were taken with a Zeiss microscope.

### Statistical analyses

All RNA sequencing data were analysed using R software (version 3.6.3). Kaplan-Meier analysis and cox analysis were used to assess the impact of CENPM on survival outcomes in ccRCC patients, and spearman correlation analysis was used to describe the correlation between CENPM expression and tumor-infiltrating lymphocytes (TILs), immune checkpoint inhibitors, the chemokines, and associated genes, with *p* < 0.05 considered statistically significant.

## Results

### CENPM mRNA expression is upregulated in ccRCC

**Fig. 1:**
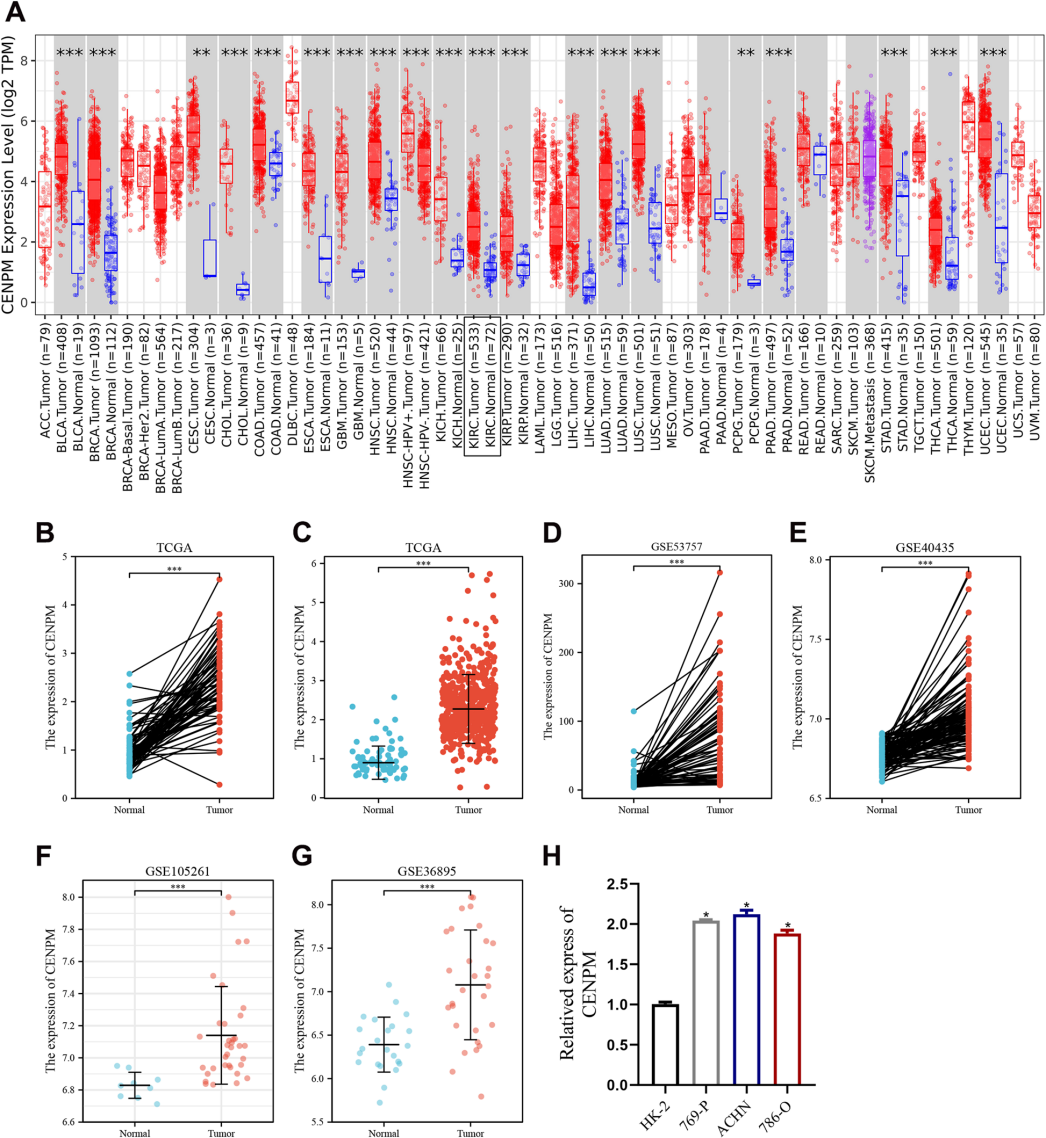
CENPM mRNA expression in ccRCC tissue samples and renal cancer cell lines. (A) Pan-cancer analysis of CENPM. (B-G) RNA sequencing data from TCGA, GSE53757, GSE40435, GSE105261, GSE36895 revealed that CENPM expression was upregulated in ccRCC samples. (H) CENPM expression was upregulated in renal cancer cell lines (769-P, ACHN and 786-O) relative to renal tubular epithelial cells (HK-2). *, *p* < 0.05, **, *p* < 0.01, ***, *p* < 0.001. CENPM, Centromere protein M. ccRCC, Clear cell renal cell carcinoma

In view of the cancer-promoting role of CENPM in a variety of human cancers, we performed a pan-cancer analysis of CENPM at the mRNA level through the TCGA database (Fig. 1A). Notably, CENPM was significantly upregulated in ccRCC tissues in both paired and unpaired samples (Fig. 1B-C). To rule out this phenomenon by chance, we confirmed the high expression status of CENPM in ccRCC tissues with four GEO datasets (Fig. 1D-G). Similarly, the up-regulation of CENPM was further confirmed in three renal cancer cell lines (Fig. 1H).

### Up-regulation of CENPM may be associated with weaker promoter methylation

**Fig. 2:**
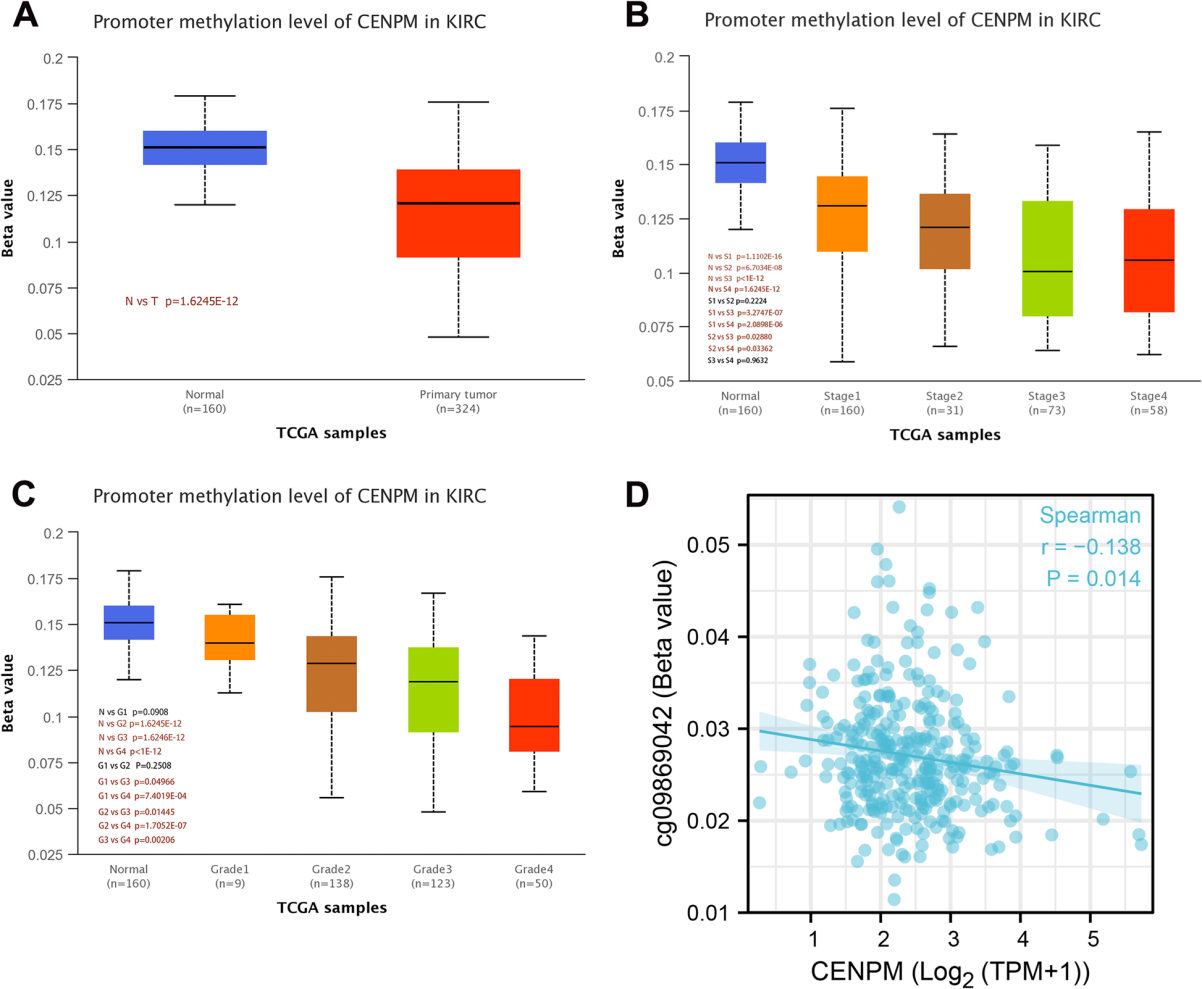
Relationship between mRNA expression of CENPM and promoter methylation levels. (A) CENPM has lower promoter methylation levels in ccRCC tissue. (B) Lower methylation levels in ccRCC samples with higher pathological stage. (C) Lower methylation levels in ccRCC samples with worse histological grading. (D) The methylation level of CENPM was negatively correlated with its mRNA expression. CENPM, Centromere protein M. ccRCC, Clear cell renal cell carcinoma

It is known that methylation of promoters can often silence gene expression, a property that could also provide a potential target for cancer therapy [20]. Therefore, based on TCGA samples, we investigated the promoter methylation levels of CENPM in ccRCC and normal tissues. The results showed that CENPM methylation levels were significantly lower in ccRCC tissues and that this trend was more pronounced in patients with more advanced pathological staging and higher histological grading (Fig. 2A-C). Also, spearman analysis demonstrated this negative correlation well (Fig. 2D).

### CENPM is associated with a more aggressive clinical profile

**Fig. 3:**
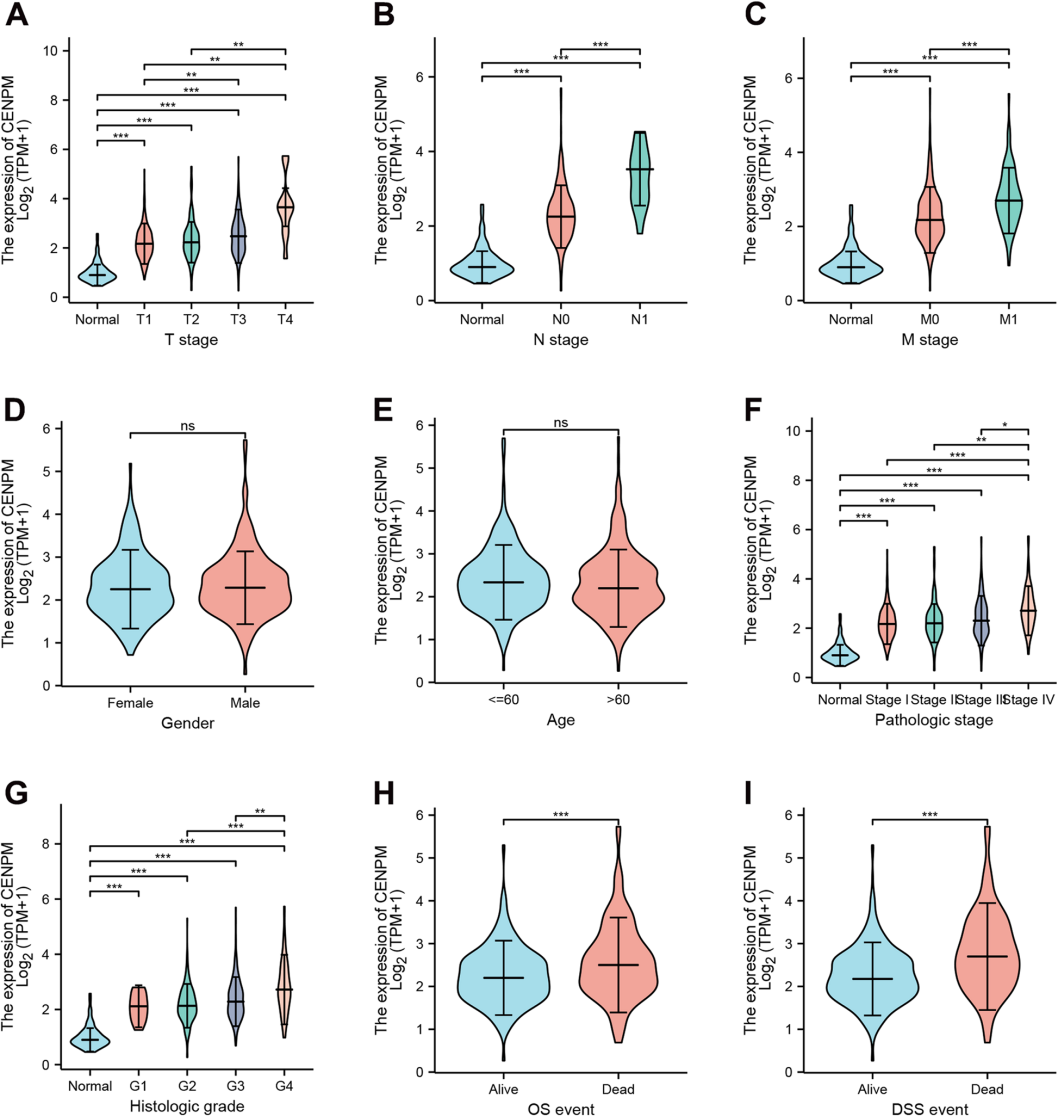
Relationship between CENPM mRNA expression and clinicopathological characteristics. Up-regulation of expression was observed in patients with high T-stage (A), lymph node metastasis (B), distant metastasis (C), high pathological stage (F), high histological grade (G) and the occurrence of fatal events (H-I); while there was no significant correlation with gender (D) and age (E). *, p < 0.05, **, p < 0.01, ***, p < 0.001. CENPM, Centromere protein M

On the basis of the upregulation of CENPM expression in ccRCC, we then explored the correspondence between its expression and clinicopathological features. As expected, CENPM expression was independent of patient age and gender, and was significantly higher in patients with more advanced clinicopathological staging, higher histological grading (Fuhrman grade) and in those who had a fatal event (Fig. 3A-I). with this evidence, we hypothesize that CENPM may be associated with the malignant phenotype of ccRCC and is detrimental to patient survival outcomes.

### The potential of CENPM as a biomarker

**Fig. 4:**
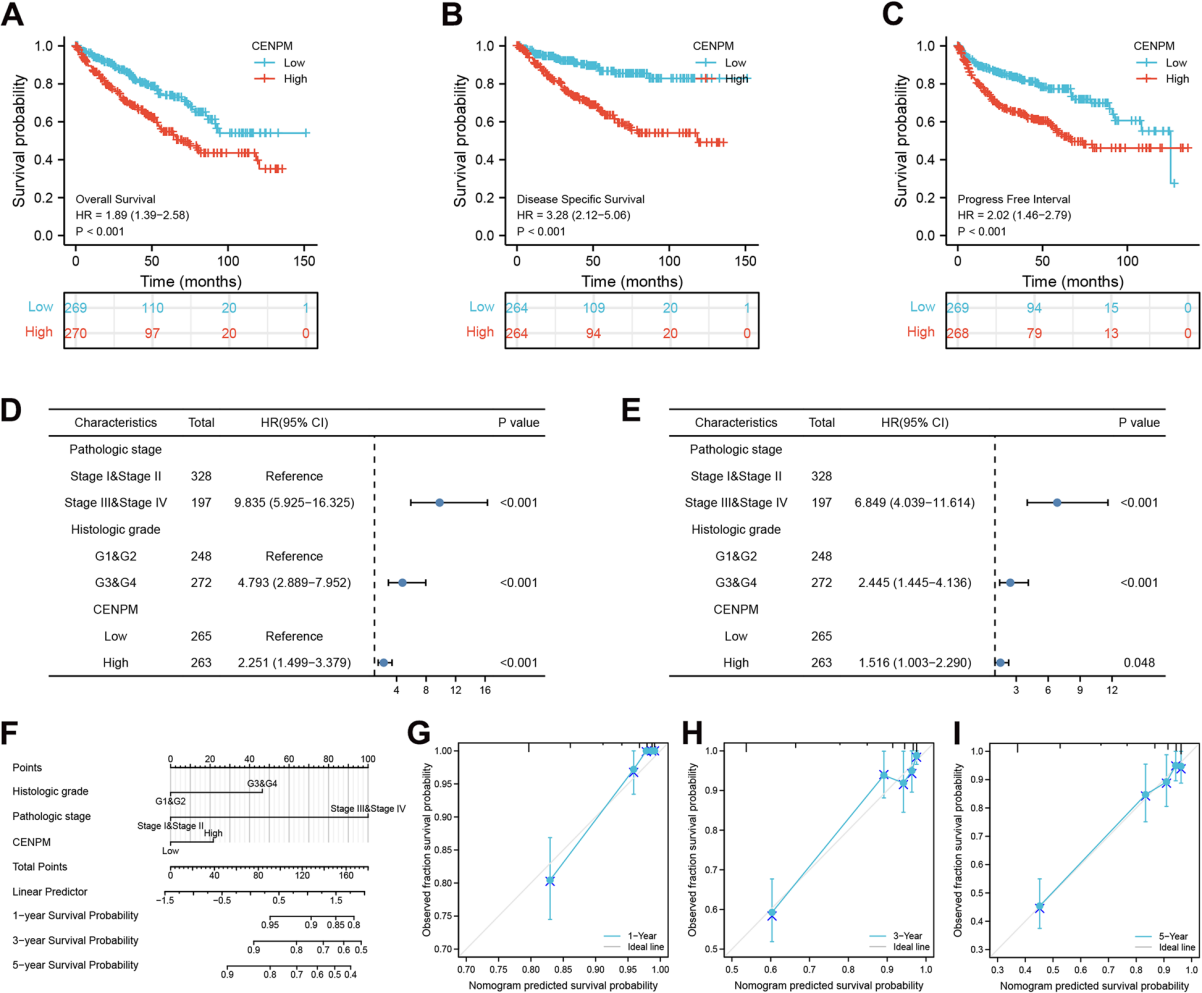
Analysis of the diagnostic and prognostic value of CENPM in ccRCC. Kaplan-Meier analysis showed that ccRCC patients with upregulated CENPM expression had significantly shorter OS (A), DSS (B) and PFI (C). Univariate (D) and multivariate (E) COX regression analyses identified CENPM as an independent prognostic factor for ccRCC patients. (F-I) Nomograms and their calibration curves constructed on the basis of independent prognostic factors in ccRCC. CENPM, Centromere protein M. OS, overall survival. DSS, disease-specific survival. PFI, progression-free interval. ccRCC, Clear cell renal cell carcinoma

As CENPM was differentially expressed in kidney cancer samples and paraneoplastic samples, we demonstrated the effect of high CENPM expression on survival outcome in ccRCC patients by means of Kaplan-Meier curves, and the results revealed that CENPM upregulation was strongly associated with worsening overall survival (OS), DSS and progression-free interval (PFI) (Fig. 4A-C). Meanwhile, we then implemented univariate (Fig. 4D) and multivariate **(**Fig. 4E) cox regression analyses and further confirmed that CENPM could be an independent prognostic factor for ccRCC. Furthermore, based on the results of the cox analysis, we further constructed Nomogram to predict 1, 3 and 5 year DSS in ccRCC patients (Fig. 4F). The C-index for evaluating its predictive efficacy was 0.803 and the calibration curve visually demonstrates the reliability of the model (Fig. 4G-I).

### Exploring the potential mechanisms of CENPM in ccRCC

**Fig. 5:**
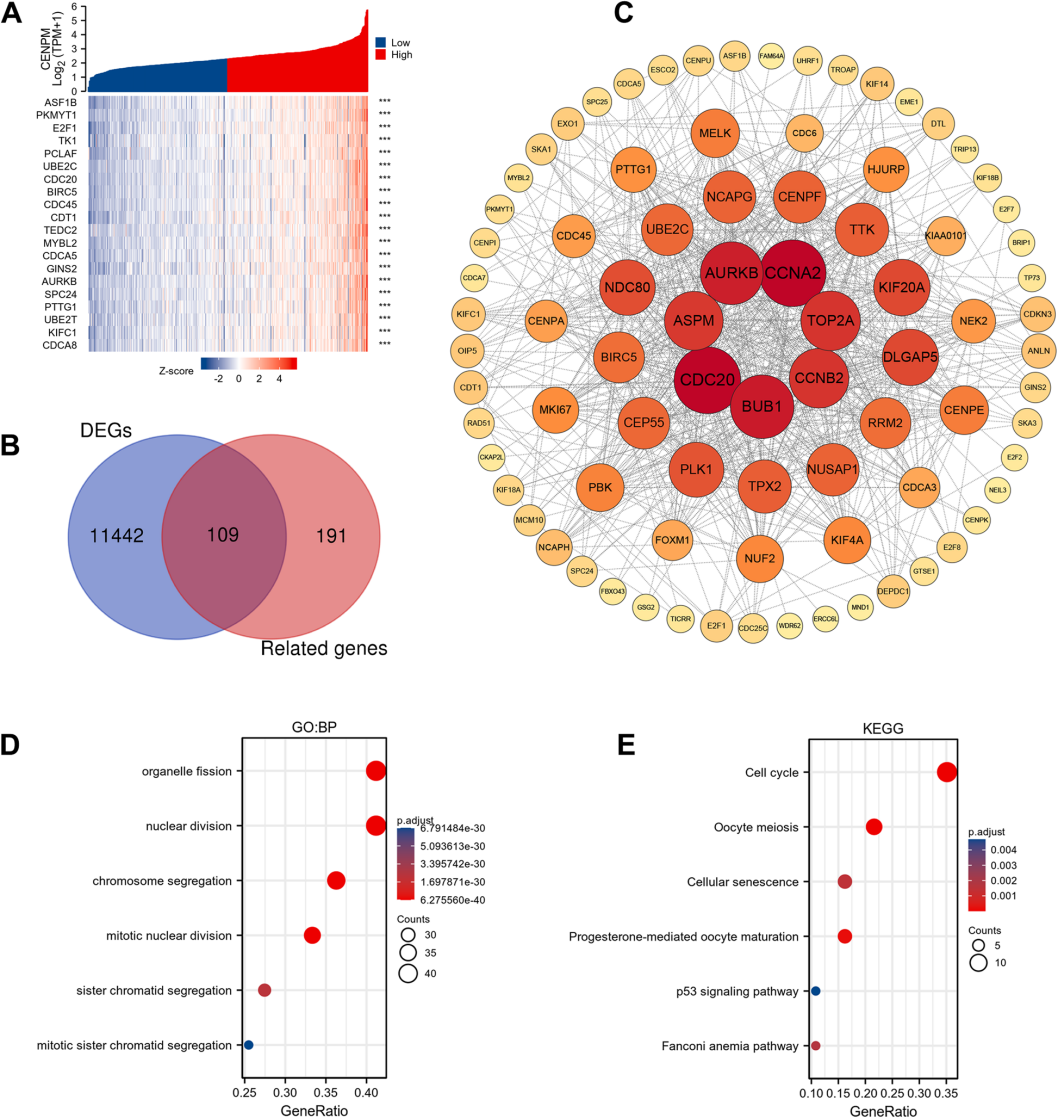
CENPM correlation gene analysis, PPI network construction, GO and KEGG analysis. (**A**) The top 20 most relevant genes for CENPM. (**B**) Intersection of related genes in CENPM and differential genes in ccRCC. (**C**) PPI network of differential genes associated with CENPM. GO (D) and KEGG (E) analysis based on CENPM-associated differential genes. PPI, Protein–protein interaction. CENPM, Centromere protein M. DEGs: differentially expressed genes. ccRCC, Clear cell renal cell carcinoma. GO, Gene Ontology. KEGG, Kyoto Encyclopedia of Genes and Genomes

By correlation analysis, we obtained the top 300 genes most associated with CENPM, and the top 20 molecules were shown by heat map in Fig. 5A. 109 of the 300 genes were differentially expressed in ccRCC (Fig. 5B), and the PPI network of these genes is shown in Fig. 5C. Based on the centrality of the nodes, we found that CCNA2, CDC20, AURKB, ASPM, BUB1, TOP2A, and CCNB2 are perhaps the most critical molecules associated with CENPM in ccRCC. In addition, based on these target genes, we performed GO and KEGG analyses, which showed that CENPM may be associated with cell division and the cell cycle in ccRCC (Fig. 5D-E). Clearly, this is similar to the findings of previous studies [6]. In parallel, we performed a potential pathway exploration through GSEA. As shown in Supplemental Fig. 1, interleukin signalling, immunomodulatory interactions between lymphocytes and non-lymphoid cells, B-cell receptor signalling, interferon signalling, cytokine-receptor interactions, cell cycle, MAPK signalling pathways, and chemokine signalling pathways were significantly enriched.

### Crosstalk between CENPM and the immune microenvironment

**Fig. 6:**
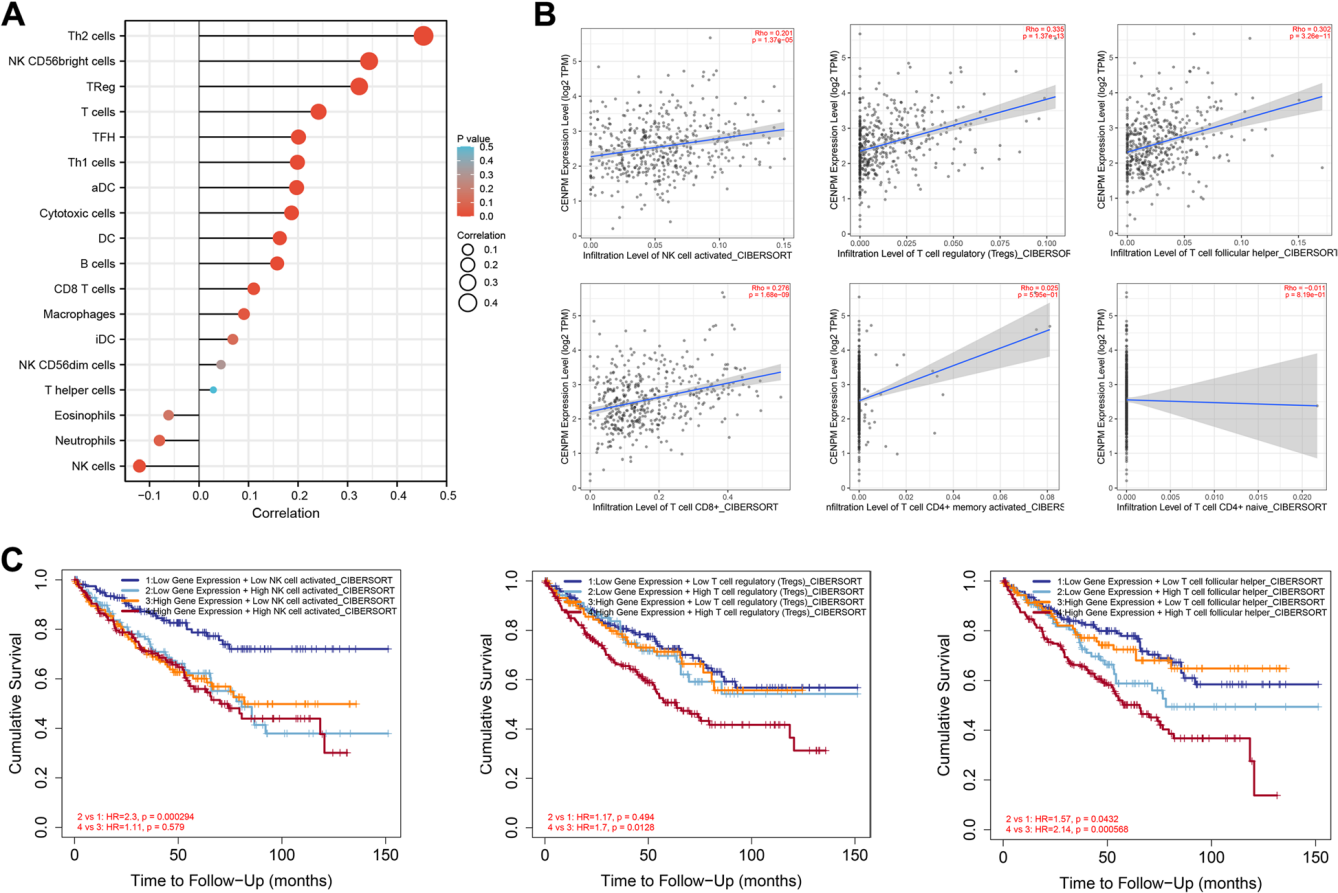
Correlation of CENPM mRNA expression with TILs in ccRCC. (**A**) TILs associated with CENPM are shown based on the ssGSEA algorithm. (**B**) TILs associated with CENPM are demonstrated based on the CIBERSORT algorithm. (**C**) Enrichment of activated NK cells, regulatory T cells and T cell follicular helpers was associated with poor OS. CENPM, Centromere protein M. TILs, tumor-infiltrating lymphocytes. ccRCC, clear cell renal cell carcinoma. OS, overall survival

Given that the GSEA results suggest that CENPM may be associated with immune infiltration in ccRCC, we first analysed the crosstalk between CENPM and TILs. Based on the ssGSEA algorithm, we found that CENPM was positively correlated with Th2 cells, T cells, T cell follicular helper, T regulatory cells, etc. (Fig. 6A). On the other hand, using the CIBERSOPT algorithm in the TIMER database, we also found that CENPM was positively correlated with activated NK cells, T cell follicular helper, T regulatory cells, and CD8+ T cells, while there was no significant correlation with CD4+ T cells (Fig. 6B). Notably, when activated NK cells, T cell follicular helper, and T regulatory cells were enriched, the OS of ccRCC patients deteriorated significantly (Fig. 6C). Taking the above information together, it is reasonable to speculate that the pro-cancer effect of CENPM may be correlated to some extent with the enrichment of unfavourable TILs.

**Fig. 7:**
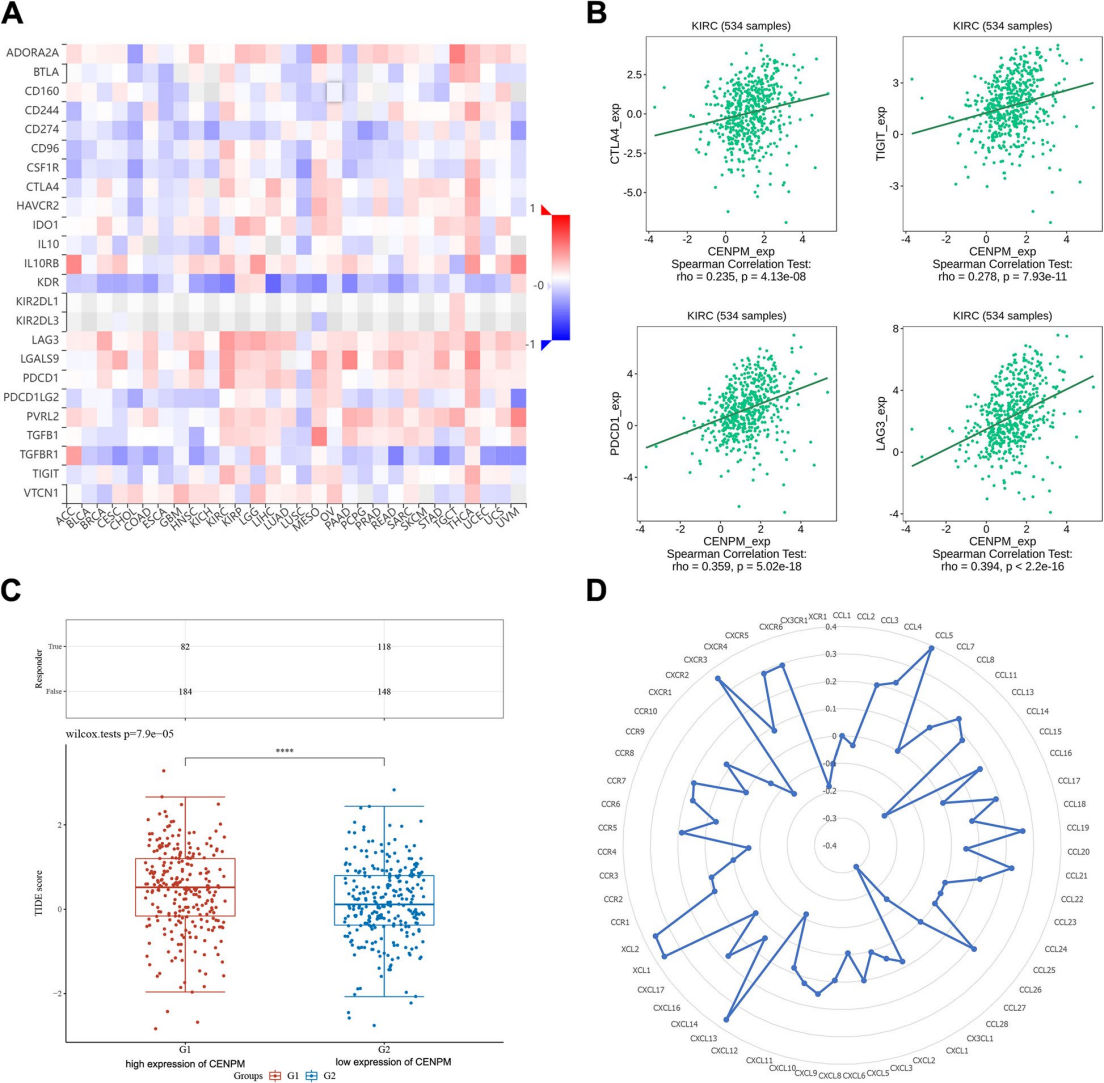
CENPM in relation to immune checkpoint genes, immunotherapy, chemokines and chemokine receptors in ccRCC. (**A**) CENPM is associated with multiple immune checkpoint genes in human cancers. (**B**) CENPM was positively correlated with CTLA4, PDCD1, TIGIT and LAG3. (**C**) Predictions based on the TIDE algorithm suggest that patients with high CENPM expression have a lower response rate to immunotherapy. (**D**) CENPM was positively correlated with CCL5, CXCL13, CXCR3, CXCR5, etc. ****, *p* < 0.0001. CENPM, Centromere protein M. ccRCC, clear cell renal cell carcinoma. TIDE, tumor immune dysfunction and exclusion

Immune checkpoints play a crucial role in the immune microenvironment in ccRCC, where they directly regulate the host’s anti-tumor immune response [21]. In this context, we have analysed the correlation between CENPM and Immunoinhibitor (Fig. 7A). Intriguingly, CENPM was significantly positively correlated with CTLA4, PDCD1, TIGIT and LAG3 (Fig. 7B), molecules that have been shown to be key immunotherapeutic targets in ccRCC [3]. The tumor immune dysfunction and exclusion (TIDE) algorithms are widely used to predict cancer immunotherapy response, with higher TIDE scores implying poorer immunotherapy outcomes [22]. As shown in Fig. 7C, the TIDE score was significantly higher and the immunotherapy response rate was significantly lower for those with high CENPM expression compared to those with low expression. Finally, the correlation of CENPM with chemokines and chemokine receptors is shown in the radar plot, where molecules such as CCL5, CXCL13, CXCR3, CXCR5 were found to be most relevant to CENPM in ccRCC (Fig. 7D).

### Preliminary validation based on IHC

**Fig. 8:**
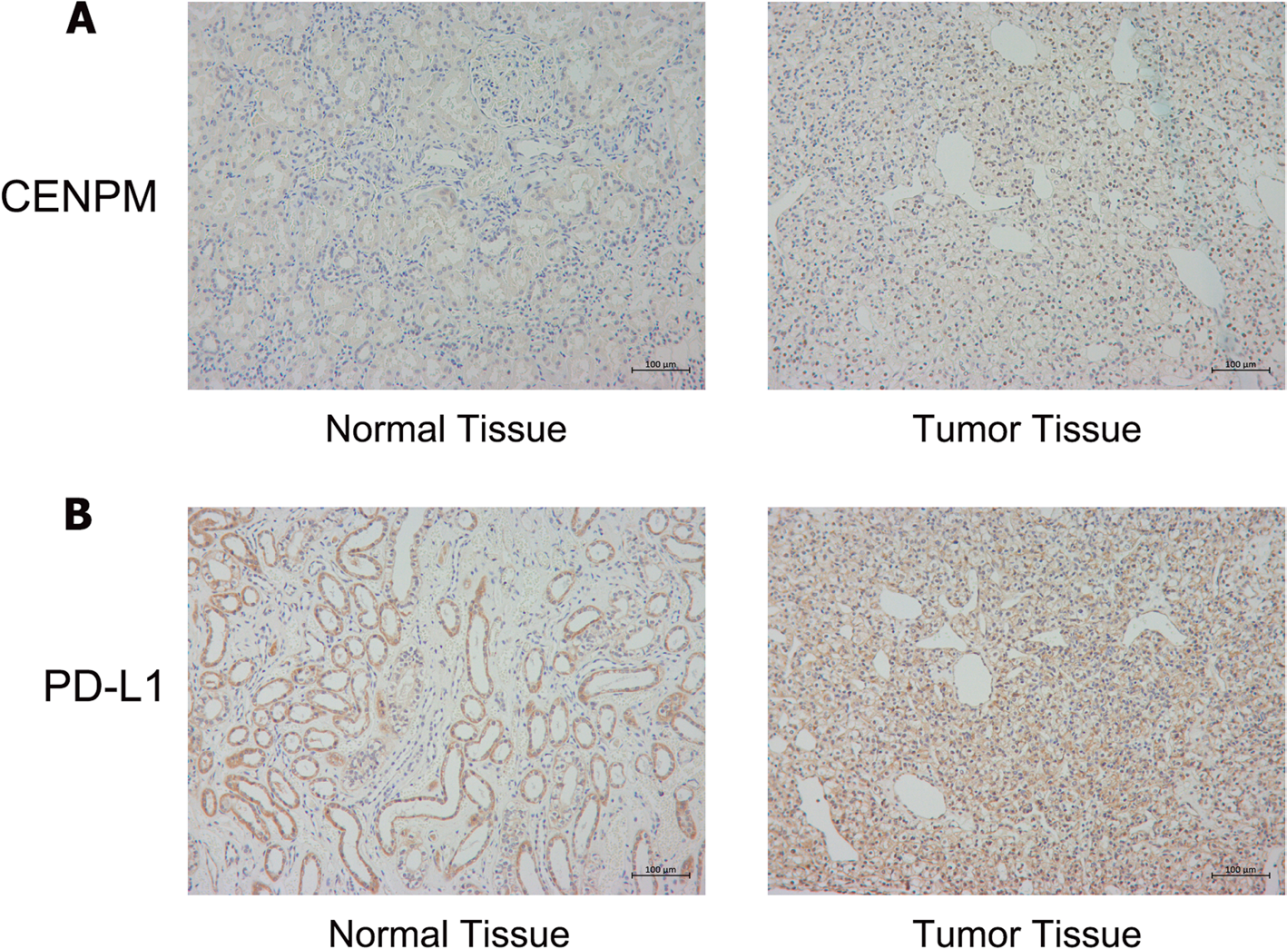
Protein expression levels of CENPM and PD-L1 in ccRCC tissues. (**A**) IHC staining of CENPM in ccRCC tissue and normal kidney tissue. (**B**) IHC staining of PD-L1 in ccRCC tissue and normal kidney tissue. CENPM, Centromere protein M. ccRCC, clear cell renal cell carcinoma. IHC, Immunohistochemistry

Given that the previous bioinformatics analysis revealed the good prognostic value of CENPM in ccRCC, we validated the expression of CENPM at the protein level using IHC. As shown in Fig. 8A, the expression of CENPM in ccRCC tissues was similarly higher than in normal paracancerous tissues, both in the cytoplasm and in the nucleus. In addition, we examined the expression of PD-L1 in ccRCC tissues considering the immunosuppressive properties of CENPM in ccRCC. Consistent with this, PD-L1 expression levels were also significantly higher in ccRCC tissues than in normal kidney tissues (Fig. 8B).

## Discussion

Centromere are regions of chromosomes that act as chromosome attachment sites for spindle microtubules in dividing cells and guide chromosome segregation in mitosis and meiosi s[23]. Centromere protein (CENP) has been identified as an autoantibody target in human disease and autoantibodies against CENPA, CENPB and CENPC have been considered relatively specific biomarkers for calcinosis, Raynaud’s phenomenon, esophageal dysmotility, sclerodactyly, and telangiectasia (CREST) syndrome [24]. In recent years, evidence for the CENP family in human cancers has been presented, including lung, breast, prostate and kidney cancers [11, 25–27]. CENPM, a component of the CENPA-nucleosome associated complex, has also been reported to be associated with liver and pancreatic cancers [8, 9]. Here, we found by bioinformatics analysis that CENPM expression was upregulated in ccRCC samples, had satisfactory diagnostic efficacy for ccRCC and was associated with worsening OS, DSS and PFI in patients. Clearly, these findings are similar to those reported in previous studies, implying that CENPM may be a promising biomarker for ccRCC.

Molecular crosstalk in the tumor microenvironment of ccRCC is known to be a complex network. In this study, we revealed some key proteins that may be closely related to CENPM by correlation analysis and PPI network construction, including: CCNA2, CCNB2, CDC20, AURKB, ASPM, BUB1 and TOP2A. Interestingly, these molecules have been shown to be associated with the malignant phenotype and poor prognosis of ccRCC in past studies [28–31]. Not only does this evidence provide a potential regulatory network, it also seems to indirectly validate the malignant function of CENPM in ccRCC.

Similar to other members of the CENP family, CENPM is thought to be involved in the regulation of cell division and the cell cycle [6]. In this paper, the results of GO, KEGG and GSEA also confirm, at least in part, the applicability of this property of CENPM in ccRCC. On the other hand, GSEA also reveals the relevance of CENPM to the immune infiltration profile in ccRCC. Indeed, in ccRCC patients, NK cell infiltration in the circulation and tumour was found to be strongly associated with an immunosuppressive phenotype [32, 33]. Regulatory T cells are one of the major immunosuppressive cell types in malignancies and a potential target for immunotherapy [34]. However, our study found that CENPM was positively correlated with these cells, highlighting the immunosuppressive phenotype to some extent. Furthermore, it is noteworthy that the enrichment of T-cell follicular helpers in ccRCC correlates with a worsening of OS (Fig. 7C), which appears to be diametrically opposed to their function in other cancers [35].

Immune checkpoint inhibitors have become the first-line treatment option for advanced ccRCC. The classical immune checkpoint inhibitors target PD-1, PDL-1 and CTLA4, while LAG3 and TIGIT are emerging promising targets [3]. Encouragingly, our study shows that CENPM positively correlates with key immune checkpoints such as CTLA-4, PD-1, TIGIT and LAG3 in ccRCC. And, the IHC results also tentatively validated this correlation (Fig. 8). This evidence predicts that CENPM may act synergistically with immune checkpoints to exacerbate the immunosuppressive state in the tumor microenvironment. Also, the TIDE score suggests lower immunotherapy response rates in patients in the CENPM high expression group. Therefore, given this evidence, combined blockade of CENPM and classical immune checkpoints may be a feasible therapeutic strategy for ccRCC in the foreseeable future.

Macrophage-derived CCL5 has been found to promote immune escape from cancer, and its co-blockade with PD-L1 enhances the anti-tumor immune response [36, 37]. In parallel, infiltration of CXCL13 CD8 + T cells in tumors may determine poor clinical outcome and immune depletion in ccRCC patients [38]. Furthermore, CXCL13 can promote ccRCC cell proliferation and migration by binding to CXCR5 and activating the PI3K/ AKT / mTOR signalling pathway [39]. CXCR3 and its ligand expression levels are also associated with prognosis, metastatic risk and tumour growth in ccRCC patients [40]. However, CENPM showed a strong correlation with these molecules, implying that its crosstalk with chemokines in tumors may also be one of the contributing factors to their malignant phenotype.

Finally, despite the systematic exploration of the prognostic value of CENPM and its relationship with the immunosuppressive phenotype in this study in ccRCC, there are still some shortcomings that need to be raised. Firstly, this study was primarily bioinformatics-based, with experimental validation limited to CENPM expression measurements at the mRNA and protein levels. Secondly, although we constructed a Nomogram to predict DSS in patients based on multiple independent prognostic factors, external validation of this could not be performed due to the lack of available datasets.

## Conclusion

Here, the first systematic evidence for CENPM in ccRCC is presented; its expression is upregulated in ccRCC and predicts a poor clinical outcome. Furthermore, CENPM expression was positively correlated with suppressive TILs, immune checkpoints, and chemokines; its crosstalk with these factors may contribute, at least in part, to the malignant phenotype in ccRCC.

## Supplementary Information

**Additional file 1:** **Supplemental Fig. 1.** Enrichment plots from GSEA. (A) Interleukin signalling pathway. (B) cell surface Interactions at the vessel wall. (C) immunomodulatory interactions between lymphocytes and non-lymphoid cells. (D) B-cell receptor signalling. (E) interferon signalling. (F) cytokine-receptor interactions. (G) MAPK signalling pathways. (H) chemokine signalling pathways. (I) cell cycle. GSEA, gene set enrichment analysis

## Data Availability

RNA-seq and clinical information for ccRCC patients are available from both the TCGA database (https://portal.gdc.cancer.gov/) and the GEO database (https://www.ncbi.nlm.nih.gov/gds/).

## Abbreviations

CENPM: Centromere protein M
ccRCC: clear cell renal cell carcinoma
TCGA: The Cancer Genome Atlas
GEO: Gene Expression Omnibus
GO: Gene ontology
KEGG: Kyoto Encyclopedia of Genes and Genomes
GSEA: gene set enrichment analysis
TILs: tumor-infiltrating lymphocytes
OS: overall survival
DSS: disease-specific survival
PFI: progression-free interval
TIDE: tumor immune dysfunction and exclusion
IHC: Immunohistochemistry.
