# Supplementary figures and images for: Upregulation of CENPM is associated with poor clinical outcome and suppression of immune profile in clear cell renal cell carcinoma
Source: Hereditas. 2023 Jan 13;160:1. doi: 10.1186/s41065-023-00262-3 (PMC9837903; doi:10.1186/s41065-023-00262-3)

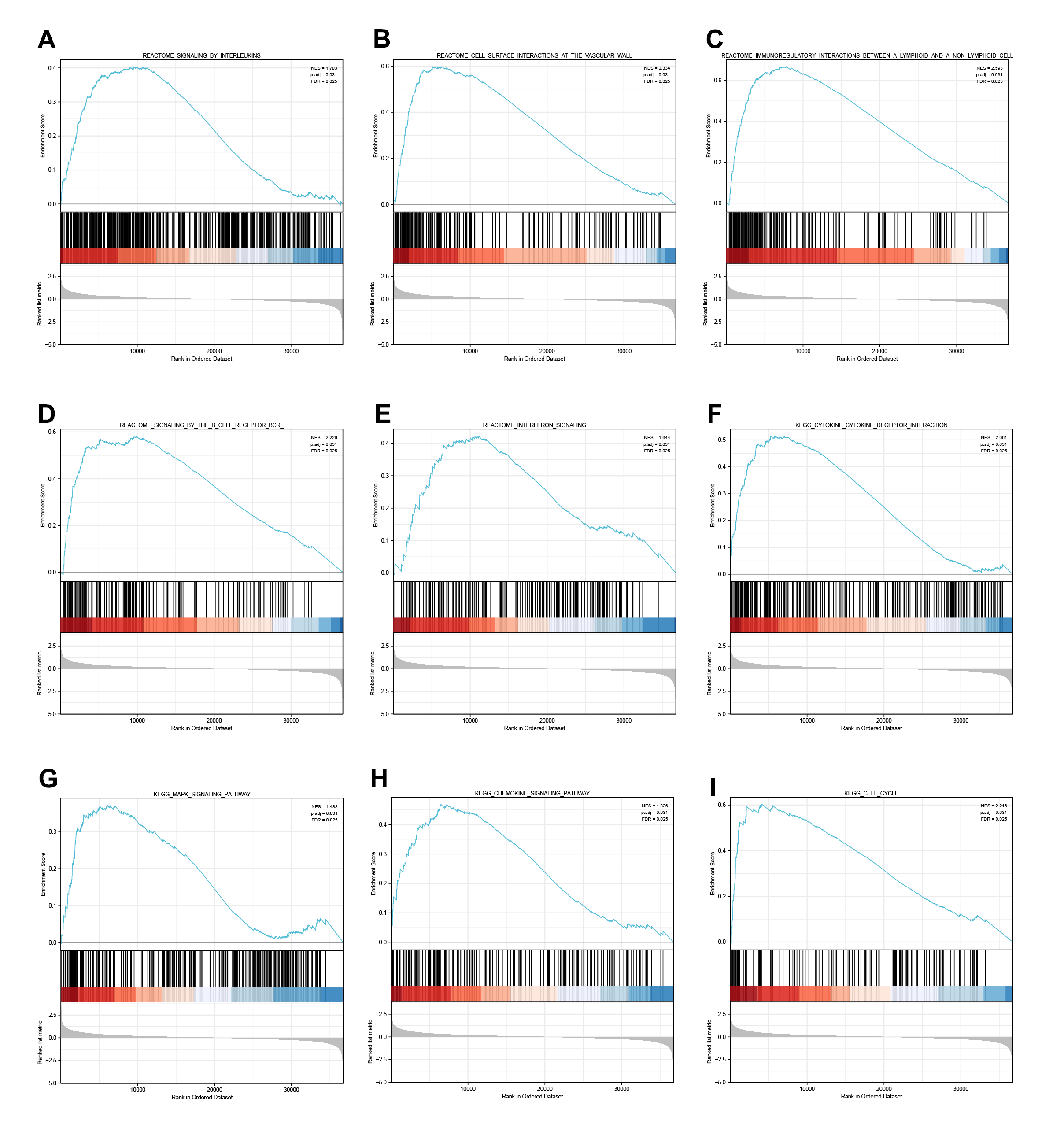

Supplement: Supplementary file 1 — Additional file 1: Supplemental Fig. 1. Enrichment plots from GSEA. (A) Interleukin signalling pathway. (B) cell surface Interactions at the vessel wall. (C) immunomodulatory interactions between lymphocytes and non-lymphoid cells. (D) B-cell receptor signalling. (E) interferon signalling. (F) cytokine-receptor interactions. (G) MAPK signalling pathways. (H) chemokine signalling pathways. (I) cell cycle. GSEA, gene set enrichment analysis [file 41065_2023_262_MOESM1_ESM.tif]
